# A rolling-gliding wear simulator for the investigation of tribological material pairings for application in total knee arthroplasty

**DOI:** 10.1186/1475-925X-9-24

**Published:** 2010-06-15

**Authors:** Berna I Richter, Sven Ostermeier, Anke Turger, Berend Denkena, Christof Hurschler

**Affiliations:** 1 Department of Orthopaedic, Laboratory of Biomaterials and Biomechanics, Medical School Hannover, Germany; 2 Department of Orthopaedic, Medical School Hannover, Germany; 3 Institute of Production Engineering and Machine Tools, Leibniz University Hannover, Germany

## Abstract

**Background:**

Material wear testing is an important technique in the development and evaluation of materials for use in implant for total knee arthroplasty. Since a knee joint induces a complex rolling-gliding movement, standardised material wear testing devices such as Pin-on-Disc or Ring-on-Disc testers are suitable to only a limited extent because they generate pure gliding motion only.

**Methods:**

A rolling-gliding wear simulator was thus designed, constructed and implemented, which simulates and reproduces the rolling-gliding movement and loading of the knee joint on specimens of simplified geometry. The technical concept was to run a base-plate, representing the tibia plateau, against a pivoted cylindrical counter-body, representing one femur condyle under an axial load. A rolling movement occurs as a result of the friction and pure gliding is induced by limiting the rotation of the cylindrical counter-body. The set up also enables simplified specimens handling and removal for gravimetrical wear measurements. Long-term wear tests and gravimetrical wear measurements were carried out on the well known material pairings: cobalt chrome-polyethylene, ceramic-polyethylene and ceramic-ceramic, over three million motion cycles to allow material comparisons to be made.

**Results:**

The observed differences in wear rates between cobalt-chrome on polyethylene and ceramic on polyethylene pairings were similar to the differences of published data for existing material-pairings. Test results on ceramic-ceramic pairings of different frontal-plane geometry and surface roughness displayed low wear rates and no fracture failures.

**Conclusions:**

The presented set up is able to simulate the rolling-gliding movement of the knee joint, is easy to use, and requires a minimum of user intervention or monitoring. It is suitable for long-term testing, and therefore a useful tool for the investigation of new and promising materials which are of interest for application in knee joint replacement implants.

## Background

During physiologic flexion and extension of the knee joint, a combined rolling and gliding motion occurs. This complex motion pattern and the incongruence of the corresponding femur and tibia condyles make the design of total knee prostheses one of the most challenging tasks in implant development. In particular, the properties of the materials responsible for the tribological pairing are of essential importance. Such materials must be biocompatible and bear mechanical load. Furthermore, they should be durable as well as exhibit very low friction and low wear rates. Wear is one of the primary factors limiting the lifetime of current knee prostheses, either directly due to material failure as a result of wear, or by aseptic loosening resulting from the accumulation of wear debris and the ensuring biological reaction [1,2]. For this reason, increasing emphasis is being placed on wear-resistant materials and surfaces to be used in joint replacement with the aim of prolonging the lifetime of knee prostheses and thus reducing or eliminating the need for revision operations. A central issue during the development of new low-wear materials and surfaces is the establishment of reliable wear testing techniques. For materials used in knee joints, the representation of a combined rolling and gliding contact is essential, since this type of motion causes different material abrasion compared to pure rolling or gliding alone [3]. Standardised methods such as Ring-on-Disc and Pin-on-Disc devices are not suitable, since they were developed for analysing pure gliding, but not a combined rolling and gliding motion [4].

Wear testing of complete total knee arthroplasty (TKA) devices for purposes of certification or product comparisons is typically carried out with commercially available knee wear simulators according to international standards such as ISO 14243. These standards seek to simulate the boundary conditions applied to a prosthetic device *in vivo *as realistically as possible in order to predict the wear behaviour of the device in clinical application. On the other hand, prosthesis geometry and design represent confounding factors contributing to wear, which cannot be isolated from the contributions of the material and surface properties alone. Moreover, complete knee-implant samples are associated with high production and acquisition costs. Thus, a TKA knee wear simulator is less suited as a materials testing advice than for product evaluation, with the aim of performing comparisons in the context of the product certification process.

To avoid the complexity and confounding effects of a knee wear simulator, as well as the over-simplification of Ring- and Pin-on-Disk approaches for purposes of materials testing, a device was constructed which includes the essential knee-like rolling-gliding motion of the knee-joint. The primary objective was to improve standardized material wear testing for applications in TKA, whereby the intended application of the device was the investigation of the effect of manufacturing processes such as machining and polishing on the surface quality, geometric tolerances, and in turn wear characteristics of biocermaic materials. Further important goals in conceiving the device were that it should be relatively inexpensive, easily transportable, capable of applying millions of loading cycles while requiring minimal user intervention, and allow wear to be measured according to standardized gravimetric methods (ASTM F1715-00, ASTM F2025-06, ISO 14243).

Herein, we describe the technical specifications, design and implementation of the device, as well as first results obtained. Data obtained from tests of the tribological pairings cobalt chrome against polyethylene (MoP), ceramic against polyethylene (CoP), and ceramic against ceramic (CoC) are reported as example applications. We hypothesized that the different wear behaviour measured with the new simulator for these well characterized material-pairings would be similar to reported values. Furthermore, tests were carried out on three further CoC tribological parings, each with a different surface roughness, and one CoC paring with convex/concave shaped running surfaces.

## Methods

### Technical concept

The physiological rolling-gliding motion of the human knee joint is represented in many typical prosthesis designs by motion occurring between the femur component and the polyethylene tibia plateau. The normalised gait cycle (ISO 14243-1) accordingly permits the following types of motion: gliding, rolling with slip, and rolling between the femur component and tibia plateau, depending on the range of motion of the knee prostheses design [5,6].

**Figure 1:**
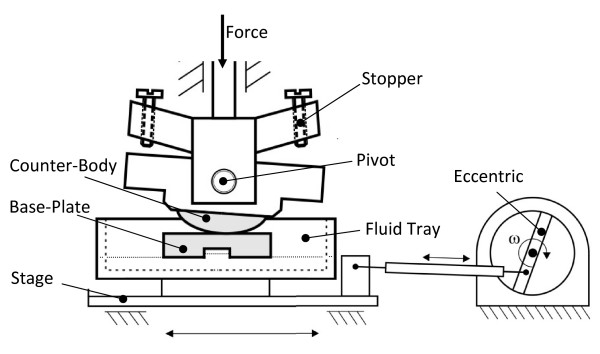
**Schematic representation of the rolling-gliding wear simulator**. The two components of the tribological pairing, the base-plate, and the cylindrical counter-body are shown in gray. Normal force is provided by a dead-weight. Sinusoidal oscillation motion of the base-plate is induced by a servo-motor and adjustable eccentric. The free rotational range of motion of the counter-body is limited by adjustable stoppers. Motion of the base-plate and counter-body occurs in a temperature-controlled bath; a second bath (not shown) is provided for an unloaded reference body.

**Figure 2:**
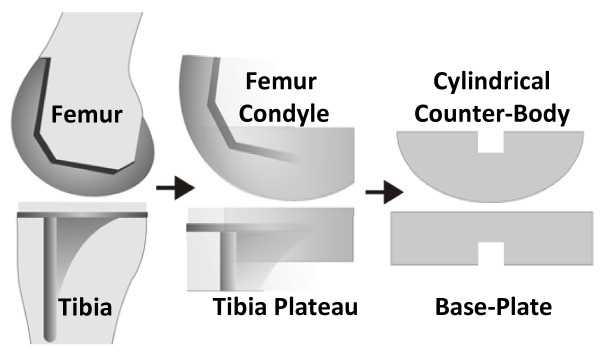
**Reduction of knee prosthesis components to specimens of simplified geometry**. Geometry of tibial and femoral knee prostheses components reduced to simple specimen geometries. The base-plate represents the tibia plateau and the cylindrical counter-body represents one femoral condyle.

Our aim was to induce these three different types of motion (pure rolling, pure gliding, rolling with slip) on simplified specimen geometries. A concept was adopted in which a base-plate was horizontally moved relative to a rotating cylindrical counter-body under axial loading (Figure. 1). In this configuration, the planar base-plate represents the tibia plateau, the so-called "inlay" of a typical knee prosthesis, and the cylindrical counter-body corresponds to one femoral condyle (Figure. 2).

**Figure 3:**
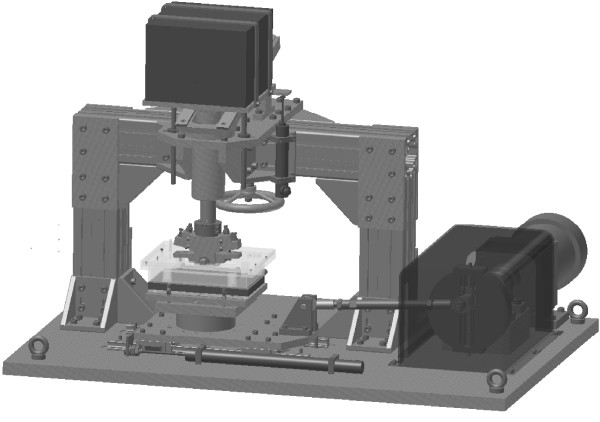
**Representation of the rolling-gliding wear simulator**. An isometric CAD representation of the rolling-gliding wear simulator without specimens is pictured. The adjustable eccentric, driven by a spur gear motor, which supplies the transversal motion of the stage equipped with linear bearings by a connected rod, is visible. The platform with weights and the hand wheel to raise the counter-body for removal of the specimens is also shown.

The "Rolling-Gliding Wear Simulator" created based on this concept consists of a horizontally oscillating stage onto which the base-plate is mounted, as well as a cylindrical counter-body which is statically vertically loaded (Figures. 1, 2 and 3). The counter-body is freely pivoted and, a rolling motion is hence induced by the driven base-plate. The rotational range of motion of the counter-body is limited by two adjustable stoppers: when the counter-body hits the first stopper whilst the stage continues in motion, a transition from rolling to gliding occurs. After a short gliding segment, the base-plate changes direction and initiates the second half of one motion cycle. At this point the cylindrical counter-body starts to roll again until hitting the second stopper inducing a second transition from rolling to gliding. The cyclical motion of the base-plate thus induces sequential cycles of rolling-gliding motion. In this manner, two different areas of wear are produced on the base-plate: a pure rolling segment in the middle of the base-plate, and two symmetrical areas of overlapping rolling and gliding adjacent to the pure rolling area. Small transition phases of rolling with slip occur between the pure rolling and overlapping rolling and gliding segments.

The material samples can be removed from the device for gravimetric wear measurement according to ASTM F1715 and ISO 14243-2, or alternatively for analysis of the wear surface profile using topological methods such as scanning electron microscopy or others [7,8].

### Mechanical Configuration

**Figure 4:**
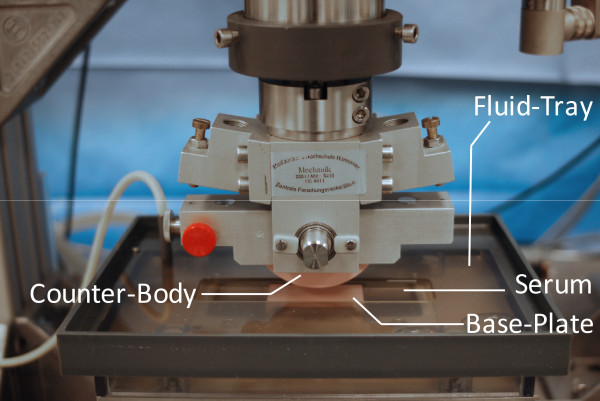
**Photograph of specimens mounted in the rolling-gliding wear simulator**. The rolling-gliding wear simulator is pictured before the initiating of testing. The cylindrical counter-body is shown in the raised position relative to the base-plate (both visible specimens are made of zirconia toughened alumina). The fluid tray is filled with serum (see methods) and the counter-body is lowered onto the base-plate for testing.

Material specimens are held in place in their two respective mounting points by a keyway and rectangular key design, with the addition of a small clamping mechanism to prevent lateral motion or dropping out of the cylindrical counter-body (Figures. 1, 4). This arrangement facilitates easy installation and removal. A platform with static weights (Figure. 3) provides constant vertical load between the counter-body and the base-plate. The platform is attached to a column which allows vertical motion only, and allows for compensation of irregularities within the articulation of the cylindrical counter-body on the base-plate.

An adjustable eccentric driven by a servo motor (Heidolph Elektro GmbH, Kehlheim, Germany, HeiDrive, Q125-0257 and D271.65) supplies the horizontal motion of the translation stage. A fluid tray, equipped with a heat-exchanger, is mounted on the translation-stage (Figures. 1, 3 and 4) into which the mounting point of the base-plate is integrated. The tray can freely tip along the translational axis to compensate for slight misalignments between the cylindrical body and the plate. This generates a uniform contact loading. A thin perspex lid covers most of the tray during testing (Figure. 4), and a peristaltic pump (not shown in figures) is calibrated to add distilled water automatically to compensate for evaporation of the test medium. Long-term-testing for well over 48 hours can thus be performed without user intervention. A second reference fluid tray (not shown in figures), also equipped with a heat-exchanger, is placed beside the test set up to use for material reference to facilitate the testing of materials which absorb liquid.

**Figure 5:**
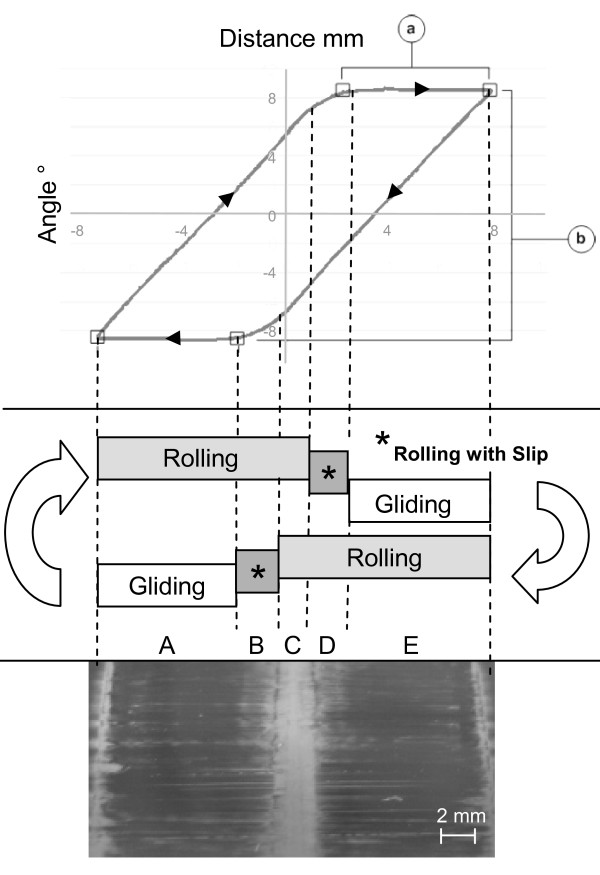
**Representation of the different loading phases**. A hysteresis-loop like loading cycle as displayed by the testing software (top), represented translational motion of the base-plate (a) and rotation of the cylindrical counter-body (b). Two phases of motion are represented, which contain elements of pure rolling and pure gliding, as well as a small transition phase of rolling with slip (middle) occurs. Because of the oscillating nature of the movement, these three different kinematic modes are superimposed. Three types of loading zones on the base-plate are hereby created: rolling and gliding, rolling with slip and rolling, and a pure rolling zone. Due to the symmetry of the system, a total of five distinct loading zones can be observed on the base-plate: A) Rolling and Gliding, B) Rolling with slip and Rolling, C) Rolling and Rolling, D) Rolling with slip and Rolling, E) Rolling and Gliding. In the scanning electron micrograph of a ceramic base-plate after 3 million cycles (bottom) the wear marks resulting from the different types of motion are visible. In the area of rolling and gliding (A and E), it is possible to identify grooves and scratches which demonstrate zones of high wear.

An electronic control module and user interface generated using LabView (Laboratory Virtual Instrument Engineering Workbench, Version 6.8, National Instruments, Austin, TX, United States) are used to manage the testing device. The software allows the user to adjust the number of testing cycles, the rotation speed of the servo motor and the amount of distilled-water added per hour. The software records the input data coming from sensors which read the linear and rotational movements, and the relative amounts of rolling, rolling and gliding, and pure gliding are graphically displayed (Figure. 5 top). A hardware counter (independent of the PC and software) provides redundancy for the prevention of data-loss in the case of a software failure.

### Testing Protocol

The wear tests are conducted under the application of a constant compressive axial load of 714 N (700 N load bars + 14 N dead load of structure) to the cylindrical counter-body. Since a single condyle is being modelled in this wear test, 714 N reflect about a half of an average normal load during stance phase of gait (0% - 66% of gait cycle) [5]. The geometry of the cylindrical counter-body is 10 mm × 21 mm (w × h) with a radius of 32 mm. The base-plate must be a minimum of 15 mm high, 15 mm wide and 30 mm long. The length of the wear track covers 15 mm, which is similar to the length of contact area on the medial compartment of the tibia during flexion [9]. To achieve a rolling-gliding, roll, rolling-gliding ratio of 1:1:1 (5 mm : 5 mm : 5 mm), the adjustable stoppers were set to allow a counter-body rotation of 17.9° (± 8.95°) which causes a rolling track distance of 10 mm using a cylindrical counter-body with a radius of 32 mm. The calculated outcome of this is a 5 mm pure rolling track and two 5 mm superimposed rolling and gliding tracks occurring on the base-plate. This correspond a rolling-gliding and rolling ratio of 2:1, which correlates with the early stages of flexion in human knee [10].

### Tribological Pairings

Altogether eight tribological pairings were tested, consisting of three different materials: Ultra High Molecular Weight Polyethylene (UHMWPE, Aesculap), Cobalt Chrome (CoCr, Aesculap), and a zirconia toughened alumina bioceramic material (BIOLOX^®^*delta*, CeramTec AG). The CoCr and PE specimens were tested as provided by the manufacturer; the ceramic specimens were tested as provided, or finished by the authors. The materials were tribologically paired as follows: a UHMWPE plate against a polished CoCr counter-body (**TP1**), a UHMWPE plate against a ceramic counter-body (**TP2**), and a ceramic plate against a ceramic counter-body (**TP3**. and **TP4**). Furthermore, three ceramic-ceramic tribological pairings with surfaces finished to different degree of roughness (R_a_) were tested: ground to R_a _= 825 nm (**TP5**), polished to R_a _= 382 nm (**TP6**), and highly polished to R_a _= 52 nm (**TP7**). Finally, another ceramic-ceramic pairing was carried out with convex and concave shaped surfaces of the base-plate and the counter-body (**TP8**), whereby the base-plate had a convex radius of 36.48 mm and the counter-body a concave radius of 37.45 mm, which represents a difference in radius of 0.97 mm. For every test series performed, a reference material sample was included, which was unloaded.

A total number of three million cycles was applied and wear was measured after 100,000, 500,000 and subsequently every millionth cycle. The wear simulator was run at 1 Hz with a mixture of 25% ± 2% calf serum and distilled water maintained 37°C ± 2°C as a test-medium, and wear was measured gravimetrically according to ASTM F1715, ISO 14243-2 and ASTM F2025 [7,8,11]. To avoid biological reactions and degradation of the testing medium, 0.2% (weight fraction, ASTM F1715-00) sodium azide was added as a biocide. When testing the UHMWPE, a material which absorbs liquid, the reference sample of the same size is placed into the second reference fluid tray as an unloaded soak control [7,11]. The gravimetrical wear was measured using a scale with an accuracy of ± 0.1 mg (ACCULAB ALC - 110.4, Sartorius Group). The gravimetrical measurement protocol includes three steps: sample preparation, weighing, and wear calculation. To prepare the testing and reference samples, they were cleaned according to a standardized protocol. To calculate the wear, the measurement results were adjusted using the reference samples and a volumetric wear rate was calculated using the density of the material. Scanning electron microscopy was used to examine the surface and wear marks on the samples.

## Results

### Wear Patterns

Because of the oscillating movement of the device, three different kinematic modes are superimposed, resulting in three types of loading zones on the base plate: rolling and gliding, rolling with slip and rolling, and a pure rolling zone. Due to the symmetry of the system, a total of five distinct loading zones were observed (Figure. 5 middle): A) rolling and gliding, B) rolling with slip and rolling, C) pure rolling, D) rolling with slip and rolling, and E) rolling and gliding.

In a scanning electron micrograph of a ceramic base-plate after 3 million cycles (Figure. 5 bottom), the wear marks resulting from the different types of motion are visible. Three distinctive main streaks (centre, left and right) and two small border lines at reversal points are revealed on the contact surface. In the areas of rolling and gliding (A and E), grooves and scratches are visible. The rolling and rolling with slip motion shows no special wearing tracks, and is therefore difficult to distinguish. On the ceramic counter-bodies, well defined smooth areas in the region of pure gliding motion were observed after 3 million cycles (not shown); however no significant gravimetric wear was registered.

### Wear Rate

**Figure 6:**
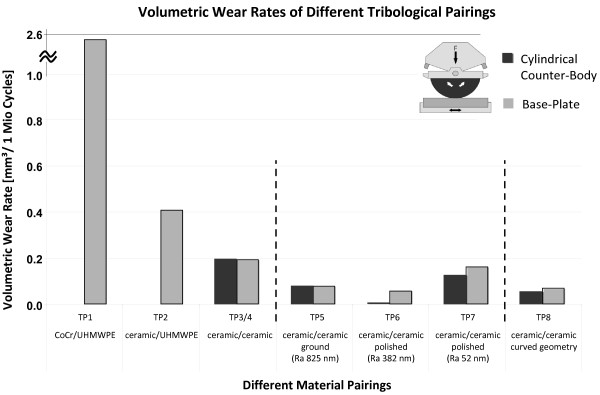
**Wear rate of different material pairings**. Total volumetric wear rate after 3 million cycles for the different material pairings investigated. Wear of the cylindrical counter-body (dark gray) and the base-plate (light gray) are illustrated. The results of the **TP3**. and **TP4**. ceramic-ceramic pairings are shown averaged; all other results mentioned are for one pairing each.

The investigated hard-soft combination of MoP (**TP1**), which is a typical pairing in knee prostheses, had an overall volumetric PE wear of 2.540 mm^3^/(10^6 ^cycles) calculated based on density of 0.943 mg/mm³ (Table. 1, Figure. 6). The wear on the CoCr counter-body was too small to detect with the scale at our disposal. The second hard-soft combination (**TP2**) using ceramic instead of CoCr shows a lower volumetric wear rate of 0.406 mm^3^/(10^6 ^cycles) for the soft base-plate. The wear of the hard ceramic counter-body was not detectable.

**Table 1:** Volumetric wear rate of different material pairings

Tribological Pairing	Number	Volumetric Wear Rate [mm^3^/1 Mio Cycles]	
Counter-Body-Base-Plate	--	Cylindrical Counter-Body	Base-Plate

CoCr-UHMWPE	TP1	--	2.5398

ceramic-UHMWPE	TP2	--	0.4060

ceramic-ceramic	TP3/4	0.1951	0.1922

ceramic-ceramic ground (Ra 825 nm)	TP5	0.0788	0.0763

ceramic-ceramic polished (Ra 382 nm)	TP6	0.0029	0.0559

ceramic-ceramic polished (Ra 52 nm)	TP7	0.1271	0.1602

ceramic-ceramic curved geometry	TP8	0.0557	0.0664

Total volumetric wear rate after 3 million cycles for different tribological pairings tested in the rolling-gliding wear simulator. No data is listed when the weight loss was too small to measure with our scale.

The average result of two hard-hard CoC material pairings of (**TP3, TP4**) show a low volumetric wear for both the base-plate, 0.192 ± 0.002 mm^3^/(10^6 ^cycles) and the counter-body, 0.195 ± 0.063 mm^3^/(10^6 ^cycles) calculated with a density of 4.37 mg/mm^3 ^for the bioceramic.

**Figure 7:**
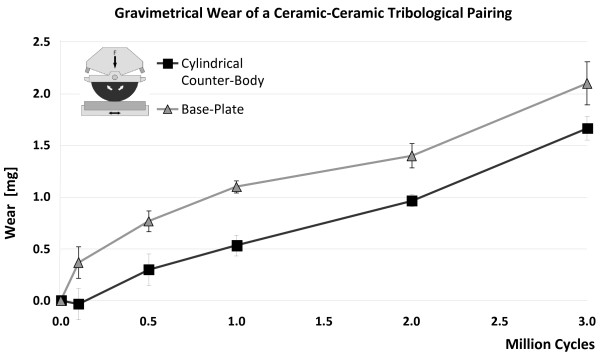
**Wear of a ceramic-ceramic material pairing in relation to the number of motion cycles**. Resulting wear of a polished (R_a _= 52 nm) ceramic-ceramic material pairing measured gravimetrically. The base-plate was run against a ceramic counter-body (**TP 5**) for three million motion cycles.

All results of the CoC tribological pairings with different surface roughness (**TP5-TP7**) are below 0.161 mm^3^/(10^6 ^cycles), (Table. 1, Figure. 6) and the curved geometry samples (**TP8**) show a low wear rate of less than 0.067 mm^3^/(10^6 ^cycles). The gravimetric-wear rate of the base-plate and counter-body of CoC material pairings displays typical patterns, whereby the base-plate exhibits an initial higher slope with a following nearly linear behaviour over three million cycles of motion (Figure. 7).

## Discussion

A testing device was designed and constructed which allows the simulation of knee-typical loading and rolling-gliding motion on material specimens of a simplified geometry. A standardized analysis of the wear behaviour of different tribological material pairings can thus be performed under conditions similar to the physiological motions and loadings of the knee joint during walking using simplified, easy to manufacture, and relatively inexpensive test specimens. Volumetric wear rates based on gravimetric measurements were presented for standard material pairings used in existing knee-prostheses (CoCr-PE, ceramic-PE), as well as for CoC material pairings with different frontal plane geometries and surface roughness. Typical wear patterns of a CoC material pairing were also described.

### Wear Testing

We observed a volumetric wear rate of the CoC pairings of less than 0.20 mm^3^/(10^6 ^cycles) which was over one order of magnitude lower than the MoP pairing at about 2.5 mm^3^/(10^6 ^cycles). The relative reduction of wear in the CoC parings compared to MoP we observed is similar to the reduction seen in total hip arthroplasty. Values reported there range from 8-35 mm^3^/(10^6 ^cycles) for MoP [12,13], and from 0.01 to 1.8 mm^3^/(10^6 ^cycles) for CoC pairing [12,14-16]. Since we reduced knee kinematics to a simplified rolling-gliding motion, our wear data cannot be directly compared with the results of wear simulators in which complete prosthesis devices are tested. Nonetheless, comparatively, the values we observed are similar to absolute values observed for complete MoP knee-prostheses, which are reported to range from about 3 to 5 mm^3^/(10^6 ^cycles) [17,18]. The relative reduction in wear reported when going from a MoP pairing, with 3.4 mm^3^/(10^6 ^cycles) to a CoP (Alumina-PE) pairing at 0.7 mm^3^/(10^6 ^cycles) [18], is similar to what we observed when comparing these two different material pairings.

Regarding the wear patterns of the CoC pairings investigated, reproducible patterns were observed on the cylindrical counter-body and base-plate. The ceramic base-plate (Figure. 5, bottom) thus typically shows small bordering lines at motion reversal points after 3 million cycles. Furthermore, the counter-body shows flat areas in the region of pure gliding, these flat areas in turn create a small edge on the counter-body surface. When the rolling movement is initiated during the shift of direction, the edge of this flat area causes contact stress on the base-plate and explains the appearance of bordering lines. This effect could be very important since it could also occur in knee prostheses components with hard-hard pairings as a result of rolling-gliding movements. As a further note, the set-up we used produces overlapping rolling-gliding areas (Figure. 5, A and 5E). In the physiological knee it is not clear if this type of overlapping occurs or not. Typically, the literature differentiates motion at the contact between rolling and gliding areas, but the degree in which these areas can shift depending on the type of motions or the presence of active muscle force is not generally described [19].

The investigation of CoC tribological pairings under the knee-like loadings and kinematics is especially interesting in the context of the realisation of low-wear CoC knee prostheses. Two interesting aspects related to the application of the CoC material pairing in knee-prostheses are the influence of geometric tolerances and surface quality on wear [20]. Geometric tolerances have an influence because they relate to the geometric congruence of parts which are in motion relative to one another (for example the inlay and femoral condyles of a knee-prosthesis). Surface quality is assumed to influence wear, because in technical applications, microstructure and surface roughness has been observed to have a positive effect on lubrication [21,22]. The effects of frontal-plane geometric tolerance as well as surface roughness on wear were thus investigated herein.

The results of preliminary experiments on the effect of surface roughness on wear (Table. 1) suggest that a highly polished ceramic surface may not be the lowest wearing surface under the knee-like conditions tested. This is an interesting observation which we plan on pursuing in further investigations. Furthermore, specimens with a curved frontal-plane geometry displayed similar wear and no fractures, despite the fact that a critical point contact load was created by difference in frontal-plane radii between the base-plate and counter-body, and that such point loadings can typically causes brittle-failure in ceramics.

### Design and Kinematics

As we have shown, the wear we observe with our device is comparable in magnitude and trend to wear-tester results of complete prosthetic devices. The device thus seems to be suitable for making comparisons between different material pairings. It should however be remembered, that the simplified boundary conditions applied (loading, kinematics) and the simplified geometry of the specimens cannot, and are not intended to represent the effect of complex design elements of knee prostheses, such as fixed or mobile inlays and or guiding or stabilizing structures. A further important consideration and possible limitation of our device is that it does not produce cross sheering motions, which are believed to be of importance in PE wear [23,24]. The integration of a cross sheering stress situation is an interesting option to which could be implemented in later generations of our device. Nonetheless, compared to other approaches such as Pin-on-Disc or Ring-on-Disc simulations, the rolling-gliding simulator provides a more suitable platform for investigating surface wear behaviour and the study of early stage component wear under knee-like loading and motion. The behaviour of different materials and the effect of surface quality and geometry (frontal plane curvature) can thus be studied without requiring the manufacture of complete knee prosthesis components.

The concept of rolling and gliding a cylindrical counter-body against a plate has also been employed in a further "wheel-on-flat" apparatus in order to analyse the wear behaviour of polyethylene material in response to increasing traction forces [3]. The results show different abrasion between pure rolling and rolling combined with gliding [4]. We therefore designed a wear simulator including these rolling-gliding movements to allow the analysis of wear behaviour in a long-term test set-up. Rolling and gliding motions are coupled in the knee joint, whereas in our setup rolling and gliding to a large degree occur successively (Figure. 5, Zones A and E). Rolling with slip occurs only in the transition zones between the pure rolling and pure gilding (Figure. 5, Zones B and D,). Initially the stoppers limiting rotation motion of the counter-body were designed with springs, which would have theoretically allowed the relative size of these transition-zones to be adjusted. This concept proved difficult to implement and was abandoned. The relative degree of rolling with slip in the transition-zones is the result of a slight structural compliance in the testing device, which cannot be adjusted. This effect nonetheless allows the representation of rolling with slip with a relatively simple device.

## Conclusions

The uncomplicated use of the testing device and the simple design of the specimens give the user the possibility of studying a wide range of technical or even natural materials with minimal user intervention over multiple million cycles of motion. The device thus allows different and new materials and or surfaces to be studied efficiently under the rolling-gliding motions similar to those to which the knee prosthesis materials are subjected to in use. The contact force and the rate of rolling and gliding can easily be changed or adjusted. By this means the behaviour of different materials and surfaces can be studied before manufacturing complete knee prosthesis components. The testing device presented herein will provide basic-research regarding issues important to the design and manufacture of ceramic-ceramic material pairings for application in TKA.

## Competing interests

The authors declare that they have no competing interests.

## Authors' contributions

BIR carried out the tests series, implemented the evaluation, analysed the data and prepared the manuscript. CH participated in the design of the study, carried out the design and construction, performed the data-analysis and helped to draft the manuscript. AT and BD supplied and prepared the specimens and analysed the geometric and surface quality. SO advised in the conception and the design of the device. All authors read and approved the final manuscript.
